# *Atractylodes* *macrocephala* Koidz. Stimulates Dermal Papilla Cells to Promote Anagen Entry Associated with GSK3β/β-Catenin Signaling

**DOI:** 10.3390/ijms27167411

**Published:** 2026-08-19

**Authors:** Su Hyeon Lee, Ji-Yoon Park, Namho Kim, Hyunwoo Park, Sung-Tae Hong, Mi Kyeong Lee, Mun-Ock Kim

**Affiliations:** 1 Natural Product Research Center, Korea Research Institute of Bioscience and Biotechnology (KRIBB), Cheongju 28116, Republic of Korea; hyeon64a3@daum.net (S.H.L.); parkgyun98@kribb.re.kr (J.-Y.P.); skagh6684@naver.com (N.K.); chung7172@kribb.re.kr (H.P.); 2 College of Pharmacy, Chungbuk National University, Cheongju 28160, Republic of Korea; 3 Department of Anatomy & Cell Biology, Department of Medical Science, College of Medicine, Chungnam National University, Daejeon 35015, Republic of Korea; mogwai@cnu.ac.kr; 4 College of Pharmacy, Chungnam National University, 99 Daehak-ro, Yuseong-gu, Daejeon 34134, Republic of Korea; 5 Department of Bioscience, University of Science and Technology (UST), Daejeon 34113, Republic of Korea

**Keywords:** *Atractylodes macrocephala*, Atractylenolides, dermal papilla cells, GSK3β/β-catenin pathway, Akt/MAPK signaling, hair-cycle progression, sesquiterpenoids

## Abstract

Although *Atractylodes macrocephala* Koidz. (AM) exhibits various pharmacological properties, its molecular effects and active constituents regarding anagen entry and physiological hair cycle progression remain uncharacterized. This study aimed to investigate the hair growth-promoting efficacy of AM and identify its bioactive compounds and underlying molecular mechanisms. We evaluated AM extract using human dermal papilla cells (DPCs) and a synchronized depilation-induced C57BL/6 mouse model, employing RT-PCR, Western blotting, immunofluorescence, and SwissADME-based pharmacokinetic profiling. AM significantly enhanced DPC proliferation and migration while upregulating paracrine factors (HGF, VEGF, and FGF7). Mechanistically, AM activated Akt, ERK, and p38 MAPKs, leading to GSK3β phosphorylation (Ser9) and subsequent β-catenin stabilization. In vivo, oral AM (60 mg/kg) markedly accelerated the telogen-to-anagen transition, increasing dermal thickness, follicle count, and hair shaft quality without systemic toxicity. Furthermore, cell-based screening and SwissADME prioritized atractylenolide I and III as the chief bioactive sesquiterpenoids driving DPC activation. In conclusion, AM and its key atractylenolides promote anagen entry and hair regrowth, in association with GSK3β phosphorylation and β-catenin stabilization, representing promising natural candidates for promoting physiological hair cycle progression.

## 1. Introduction

Hair loss, or alopecia, is a chronic dermatological condition that significantly impacts the psychological well-being and quality of life of millions of individuals worldwide. Beyond its cosmetic implications, the hair follicle is a highly sophisticated, self-renewing organ that serves as an excellent model for studying tissue regeneration, stem cell biology, and complex cellular communication [1]. The hair follicle undergoes continuous, life-long cycling through three distinct phases: anagen (active growth), catagen (regression), and telogen (quiescence). At the physical base of each hair follicle lies the dermal papilla. Composed of specialized mesenchymal cells, the dermal papilla cells (DPCs) function as the command center of the follicular microenvironment [2,3]. DPCs dictate follicle cycling, particularly the crucial transition from the quiescent telogen phase to the regenerative anagen phase, by secreting paracrine signaling molecules—such as hepatocyte growth factor (HGF), vascular endothelial growth factor (VEGF), and fibroblast growth factor 7 (FGF7)—which stimulate the proliferation and differentiation of surrounding follicular epithelial stem cells.

Currently, clinical therapeutic options for managing alopecia are highly restricted. The only two drugs approved by the United States Food and Drug Administration (FDA) are finasteride and minoxidil. Finasteride functions systemically as a 5α-reductase inhibitor to lower levels of the androgen dihydrotestosterone (DHT) [4], whereas minoxidil acts topically as a potassium channel opener to promote vasodilation and prolong the anagen phase [5]. Despite their widespread use, both treatments are hampered by notable limitations: their therapeutic efficacy varies greatly among patients, hair loss resumes upon discontinuation, and they carry risks of undesirable adverse effects, including sexual dysfunction and post-finasteride syndrome [6]. This clinical gap has driven significant research into plant-derived natural products. Botanical extracts and phytochemicals offer a promising alternative, with several key studies demonstrating that active molecules from *Polygonum multiflorum* [7], *Houttuynia cordata* [8], and *Panax ginseng* [9] can stimulate hair follicle regeneration by activating evolutionary conserved developmental pathways, most notably the Wnt/β-catenin signaling cascade.

Within the field of hair biology, a prominent scientific debate exists regarding the optimal pharmacological strategy: the “single-target” versus “multi-target/synergy” hypothesis. Classical pharmaceutical pipelines favor the identification of a single “silver bullet” molecule designed to act on a single receptor or enzyme, minimizing potential off-target effects. However, hair follicle cycling is controlled by a highly redundant and interconnected network of intracellular kinases. In this context, single-target approaches often fail to achieve robust clinical efficacy or induce compensatory bypass pathways. Conversely, the multi-target hypothesis argues that crude botanical extracts, consisting of diverse families of secondary metabolites, can simultaneously modulate parallel signaling nodes (such as the Akt, ERK, and p38 MAPK pathways) to generate a more integrated, robust biological response without causing severe systemic toxicity [10,11]. Understanding how complex natural extracts coordinate these multi-target networks at a molecular level remains a major focus of modern molecular medicine.

A highly promising yet uncharacterized candidate in this arena is *Atractylodes macrocephala* Koidzumi (AM), a perennial medicinal herb widely utilized in traditional East Asian medicine. While AM is well-recognized for its antioxidant, anti-inflammatory, and gastrointestinal benefits [12,13,14,15,16]—which are primarily attributed to its rich concentration of bioactive sesquiterpenoids—its potential therapeutic effects on hair biology and the specific molecular mechanisms involved have not yet been evaluated. Given its excellent safety profile and official recognition as an edible food-grade material, AM represents a highly viable candidate for oral phytotherapeutic development.

The main aim of this study was to systematically investigate the hair growth-promoting potential of *A. macrocephala* rhizoma extract (AM) and identify its primary bioactive constituents and molecular targets using human DPCs and a synchronized in vivo C57BL/6 mouse model. These findings provide molecular insights into the effects of AM and its key active sesquiterpenoids, supporting further investigation of AM as a candidate for modulating physiological hair-cycle progression. At the molecular level, we elucidated that AM-driven DPC activation is achieved by stimulating the Akt/MAPK (ERK, p38) pathways, which converge to induce the inhibitory phosphorylation of GSK3β (Ser9), thereby stabilizing β-catenin and upregulating crucial hair growth paracrine factors (HGF, VEGF, and FGF7). These findings provide the first concrete molecular evidence establishing AM and its key sesquiterpenoids as potent, targeted phytotherapeutic agents for anagen entry and hair regrowth.

## 2. Results

### 2.1. AM Enhances Proliferation and Upregulates Key Hair Growth-Associated Factors in DPCs

**Figure 1:**
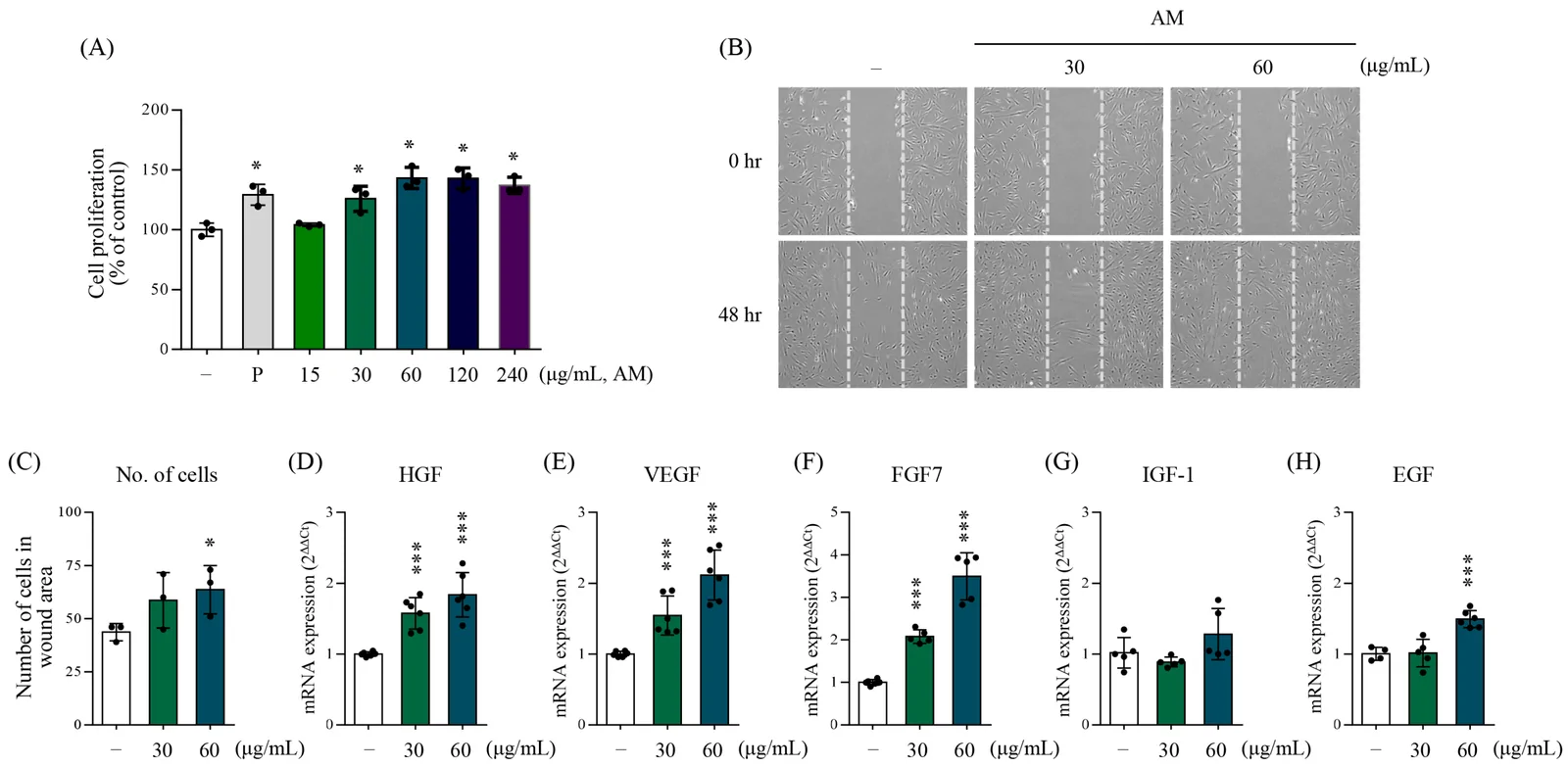
AM-induced proliferation, migration, and growth factor expression in DPCs. (**A**) DPC viability was assessed via CCK-8 assay after treatment with AM (15–240 μg/mL) for 48 h under 1% growth factor conditions (Control: 1%; Positive control: 4%). (**B**,**C**) Representative images and quantification of DPC migration following wound healing assays and 48-h AM incubation. (**D**–**H**) RT-PCR analysis of hair growth-associated mRNA levels (HGF, VEGF, FGF7, IGF-1, and EGF) after 48 h of AM treatment. Statistical significance (* *p* < 0.05, *** *p* < 0.001) is indicated relative to the control group (1% growth factor).

To investigate the effect of AM on proliferation and migration, CCK-8 and wound healing assays were conducted under growth factor–deficient (1%) conditions. AM (15, 30, and 60 μg/mL) increased DPC viability in a dose-dependent manner, reaching an optimal effect at 60 μg/mL before plateauing at higher concentrations (120 and 240 μg/mL), indicating that while AM loses its pro-proliferative activity at these threshold levels, it does not induce acute cellular cytotoxicity (Figure 1A). Similarly, AM significantly enhanced DPC scratch closure after 48 h, with the most pronounced effects observed at 30 and 60 μg/mL (Figure 1B,C). Since this assay was conducted over 48 h, this closure likely reflects a combination of enhanced cellular motility and proliferative activity, demonstrating its overall role in promoting DPC activation.

The expression of hair growth-associated factors was evaluated via RT-PCR. AM (30 and 60 μg/mL) selectively upregulated mRNA levels of HGF, VEGF (over 2-fold), and FGF7 (over 3-fold) at the highest concentration (Figure 1D–F). Conversely, AM did not significantly affect IGF-1 or EGF expression (Figure 1G,H). These results indicate that AM preferentially stimulates the expression of key paracrine factors involved in hair growth, including HGF, VEGF, and FGF7, while exerting minimal effects on IGF-1 and EGF.

### 2.2. Akt, ERK and p38-Mediated GSK-3β Inactivation Is Accompanied by AM-Induced DPC Proliferation

**Figure 2:**
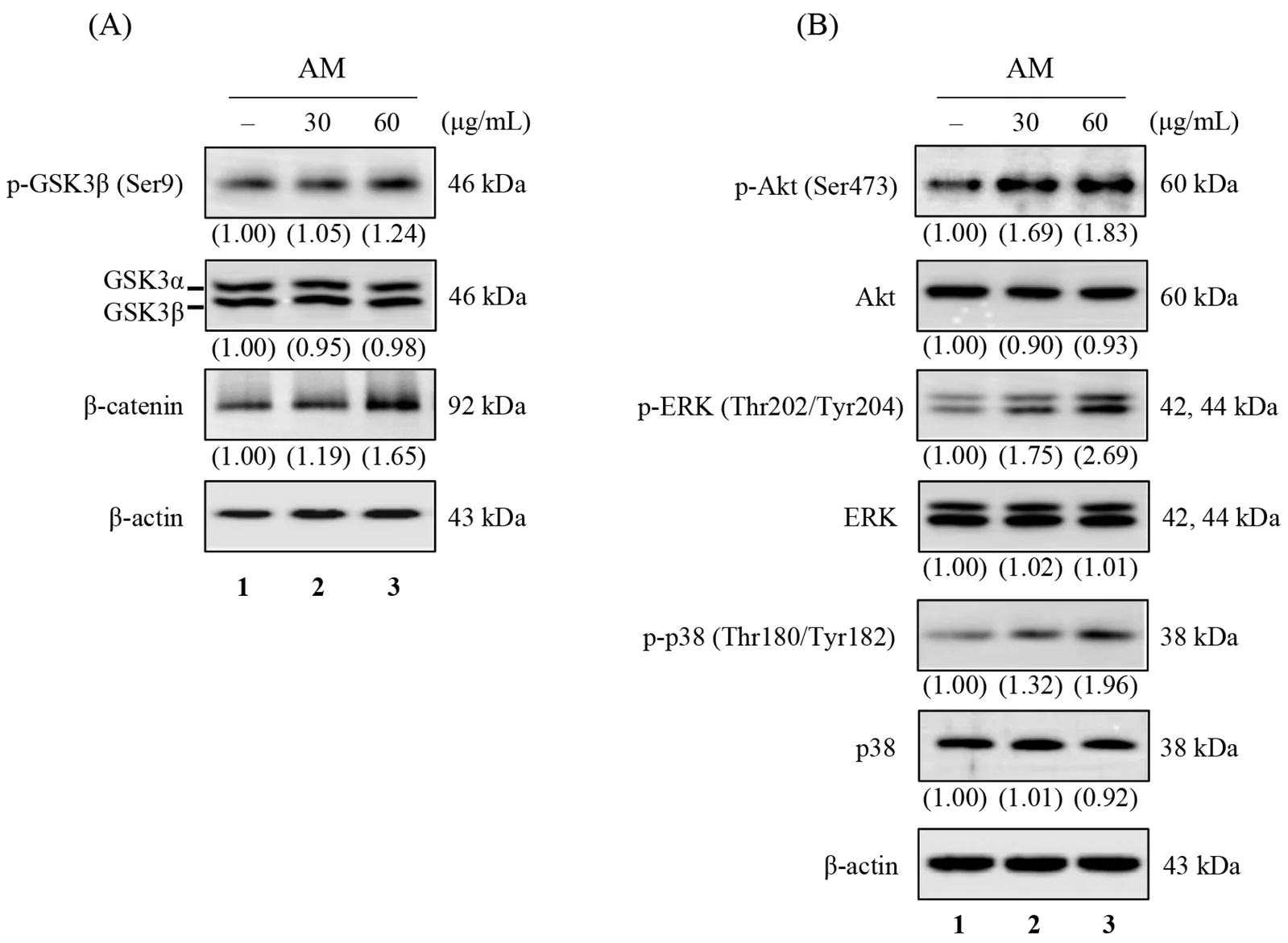
GSK3β/β-catenin signaling and upstream kinase activation in AM-treated DPCs. (**A**) Western blot detection of phosphorylated (Ser9) and total GSK3β, and β-catenin in AM-treated DPCs. (**B**) Western blot analysis of phosphorylated and total Akt, ERK, and p38, with β-actin as a loading control. Band densities are quantified numerically at the bottom of the image.

To investigate the molecular mechanisms of AM, we examined the GSK3β/β-catenin axis and its upstream kinases. AM treatment (30 and 60 μg/mL) markedly increased GSK3β phosphorylation at Ser9 and β-catenin protein abundance without altering total GSK3β levels (Figure 2A). Because Akt, ERK, and p38 are well-established upstream regulators of GSK3β inactivation—where Akt primarily mediates Ser9 phosphorylation, PKA and RSK1 can also contribute, and ERK and p38 induce additional inhibitory phosphorylation events at Thr43 and Thr389 (Thr390 in rodents), respectively [17,18,19,20]—we next assessed whether AM activates these signaling molecules. AM increased the phosphorylation of Akt (Ser473), ERK (Thr202/Tyr204), and p38 (Thr180/Tyr182) while total protein levels remained unchanged (Figure 2B). These findings indicate that AM activates multiple upstream pathways (Akt, ERK, and p38) that converge to promote GSK3β inactivation, thereby enhancing β-catenin stabilization in DPCs.

### 2.3. Hair Growth Stimulatory Effect of AM in a Time-Synchronized Depilation Model

**Figure 3:**
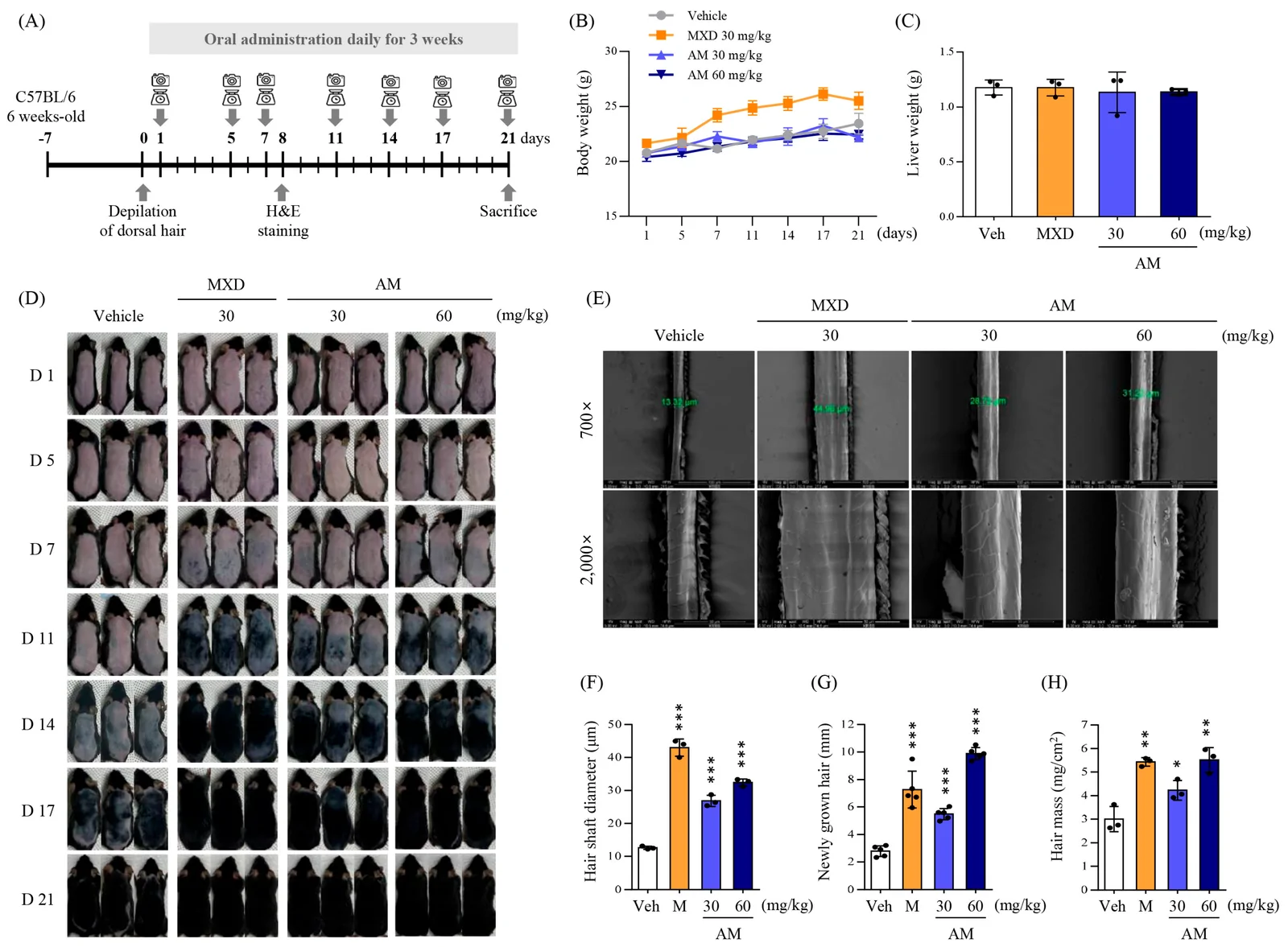
Hair growth-promoting effect of AM in a synchronized depilation mouse model. (**A**) Experimental scheme: 7-week-old C57BL/6 mice were depilated and orally administered AM (30/60 mg/kg) or minoxidil (30 mg/kg) daily for 3 weeks. (**B**,**C**) Monitoring of body weight and liver mass to evaluate systemic toxicity and hepatotoxicity. Each dot represents an individual animal. (**D**–**H**) Documentation of hair regrowth via photography and quantitative analysis of hair shaft diameter (SEM), length (caliper), and mass. Statistical significance (* *p* < 0.05, ** *p* < 0.01, *** *p* < 0.001) is indicated relative to the vehicle group.

To evaluate in vivo hair growth potential of AM, we employed a time-synchronized depilation model in 7-week-old C57BL/6 mice [21]. Mice received oral AM (30 or 60 mg/kg) or minoxidil (30 mg/kg) daily for 3 weeks (Figure 3A). Under the experimental conditions used, no overt changes in body weight or liver mass were detected, suggesting that AM treatment was well-tolerated over the 3-week study period (Figure 3B,C). Visual monitoring revealed pronounced skin pigmentation by Day 7 and substantial hair coverage by Day 14 in the AM 60 mg/kg and minoxidil groups (Figure 3D). Quantitative analysis showed that AM (60 mg/kg) markedly increased hair shaft diameter (2.8-fold), length (3.6-fold), and mass (1.8-fold) compared to vehicle controls (Figure 3E–H). These improvements in hair quality were comparable to or exceeded the effects of minoxidil. Collectively, these data demonstrate that oral administration of AM effectively promotes dose-dependent hair regrowth and enhances hair thickness and length without systemic toxicity.

### 2.4. Histological Analysis Reveals Enhanced Anagen Progression and Dermal Thickening Following AM Administration

**Figure 4:**
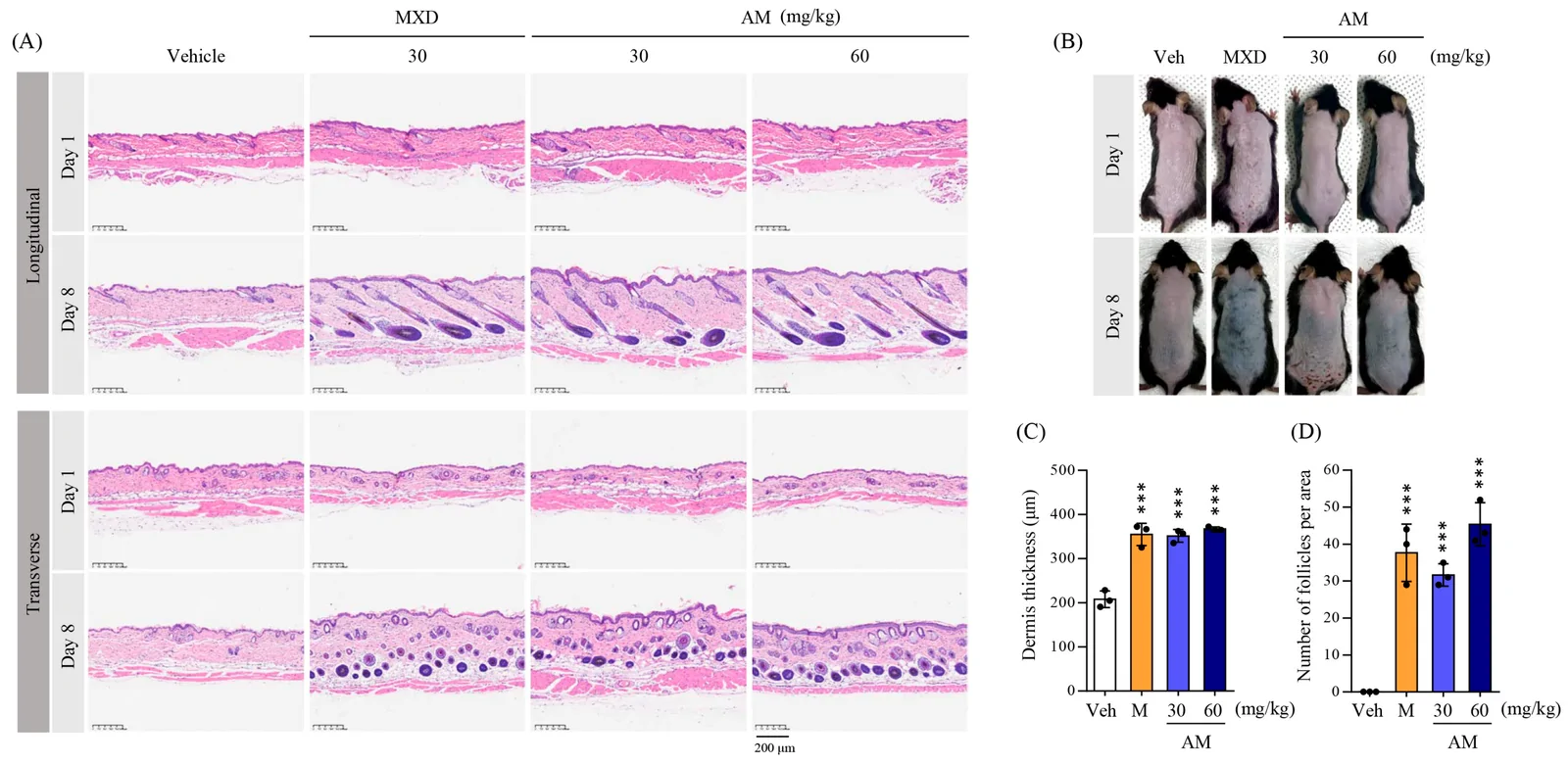
Histological evaluation of follicular regeneration and dermal structure following AM administration. (**A**,**B**) H&E staining of longitudinal/transverse sections and representative skin tissue images at Day 1 and Day 8 post-depilation. (**C**,**D**) Quantitative analysis of dermal thickness and hair follicle counts from Day 8 sections. Statistical significance (*** *p* < 0.001) is indicated relative to the vehicle group. Each dot represents an individual animal.

To analyze structural changes, H&E staining was performed on dorsal skin sections from mice treated with vehicle, minoxidil, or AM (30 and 60 mg/kg). At Day 1 post-depilation, all groups exhibited synchronized telogen follicles and thin dermis, reflecting a quiescent hair cycle (Figure 4A). By Day 8, significant morphological changes occurred in the AM and minoxidil groups, featuring marked dermal thickening and mature anagen follicles with enlarged hair bulbs extending into the subcutis (Figure 4A,B). This thickening is characteristic of anagen induction, driven by matrix deposition and follicle extension. Conversely, vehicle-treated mice largely failed to initiate anagen entry, with follicles remaining in a regressed state. Quantification revealed approximately 1.5-fold increases in dermal thickness for all treated groups relative to controls (Figure 4C). Follicle counting further supported robust activation: while the vehicle group showed no active anagen entry, AM (60 mg/kg)-treated mice exhibited an average of 42 follicles, comparable to the minoxidil group (38 follicles) (Figure 4D). Collectively, these histological findings demonstrate that AM promotes telogen-to-anagen transition and follicular maturation, corroborating the observed external hair growth phenotypes.

### 2.5. AM Enhances β-Catenin Accumulation and Proliferative Activity in Hair Follicles

**Figure 5:**
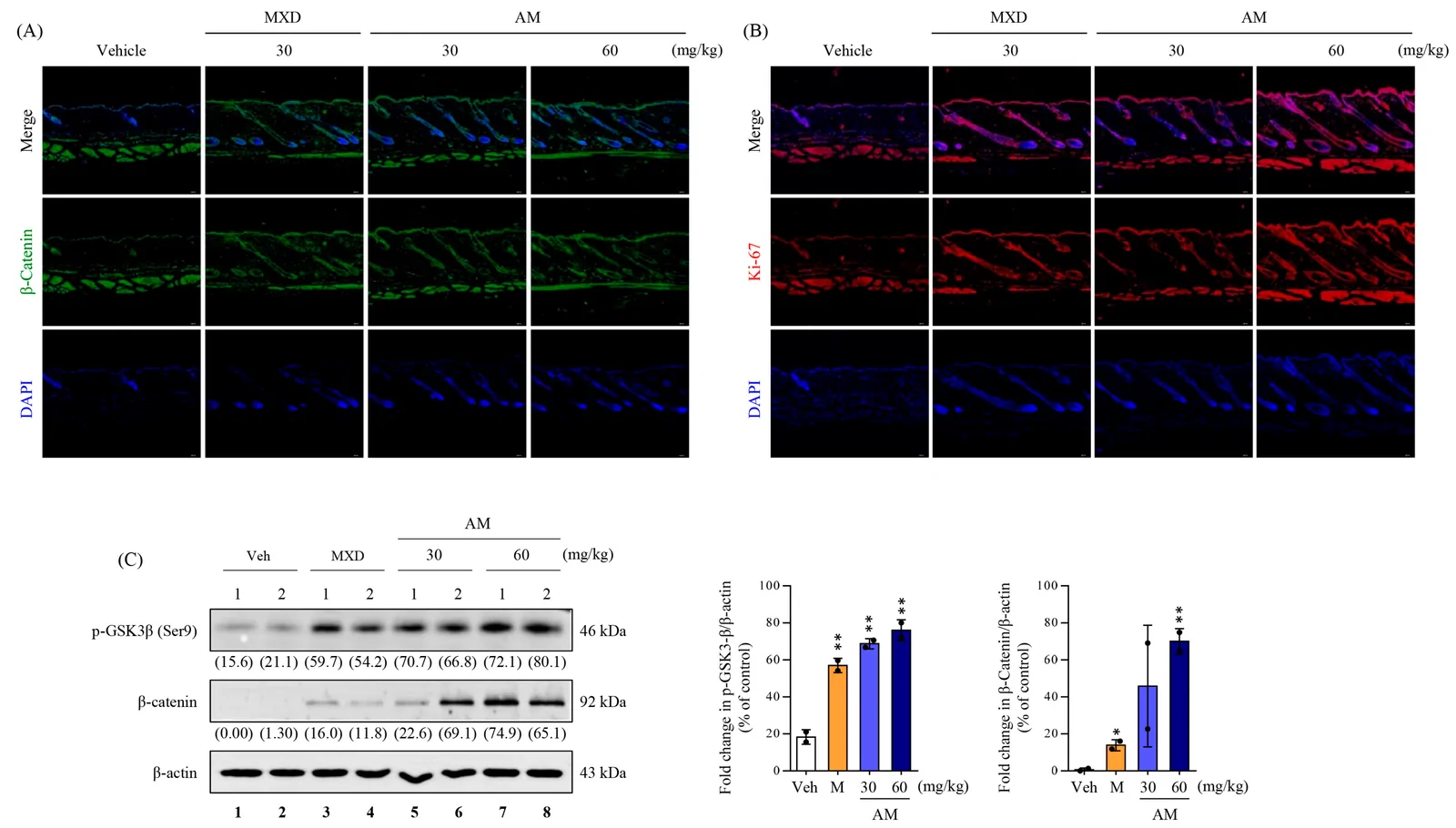
β-catenin accumulation and proliferative activity in AM-treated skin. (**A**) Immunofluorescence of β-catenin localization in the hair bulb and outer root sheath on Day 8 post-depilation. (**B**) Ki-67 staining to detect proliferative cells within the hair matrix. (**C**) Western blot of phospho-GSK3β and total β-catenin from skin lysates, with β-actin as a loading control. Band densities are quantified numerically at the bottom of the image. Statistical significance (* *p* < 0.05, ** *p* < 0.01) is indicated relative to the vehicle group.

To elucidate the molecular mechanisms of AM-induced regeneration, immunofluorescence and Western blot analyses were performed on Day 8 skin sections. AM (30–60 mg/kg) significantly increased β-catenin abundance in the hair bulb and outer root sheath (Figure 5A), and heightened Ki-67 proliferative activity in the hair matrix compared to vehicle controls (Figure 5B). These findings were corroborated by Western blotting, showing robust GSK3β phosphorylation (Ser9) and increased β-catenin levels (Figure 5C). Collectively, AM enhances β-catenin signaling and follicular proliferation within the hair matrix compartment, providing molecular support for accelerated anagen progression in vivo. However, while these markers indicate robust transient amplifying cell activity, further studies using compartment-specific stem cell markers are required to determine the precise involvement of bulge stem cells.

### 2.6. Identification and Physicochemical Evaluation of Compounds Contained in AM

**Figure 6:**
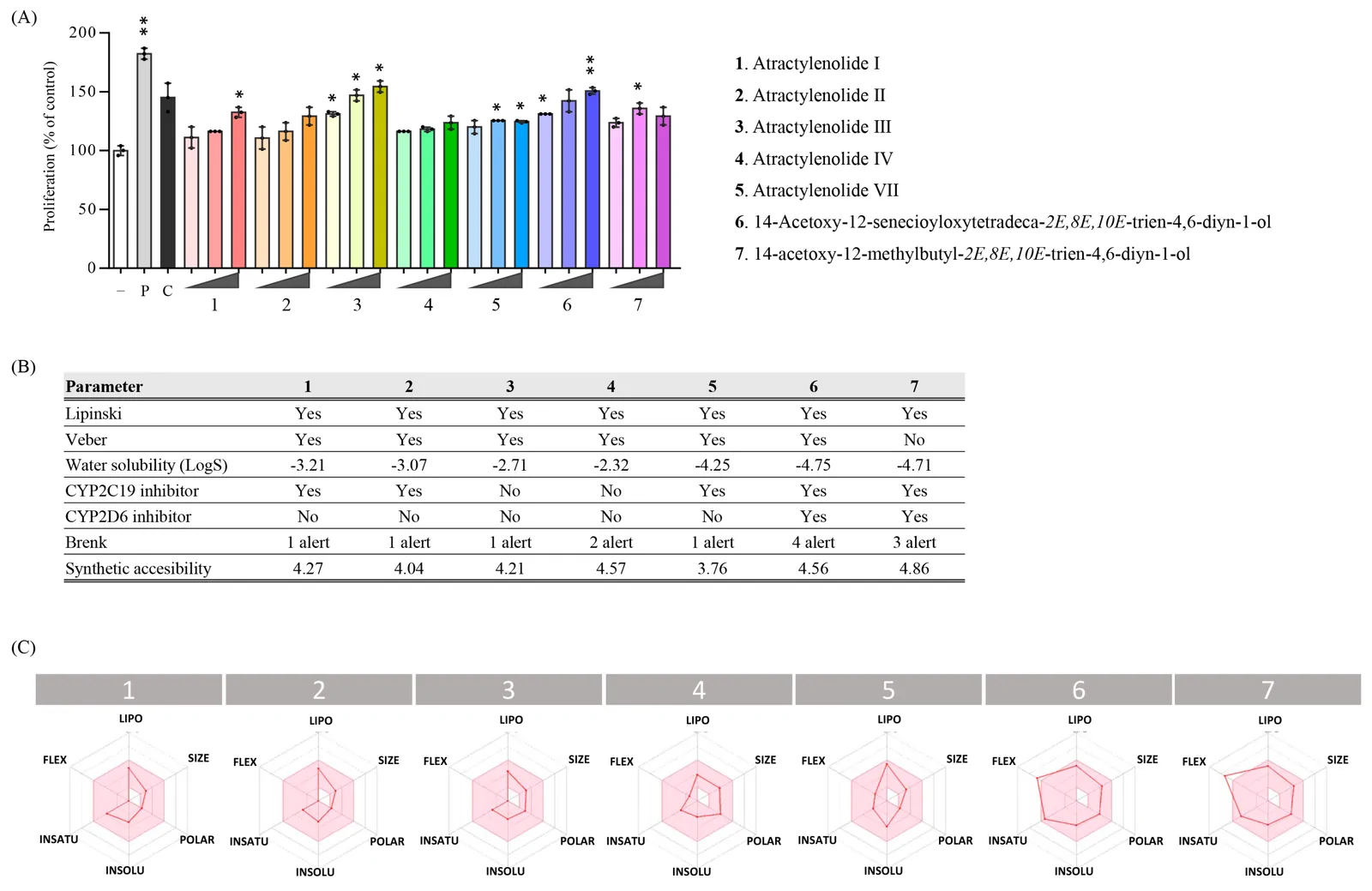
Physicochemical properties and proliferative effects of isolated compounds (**1**–**7**). (**A**) DPC proliferation following treatment with compounds **1**–**7** (20–80 μM) for 48 h under 1% growth factor conditions (C: positive control). (**B**) Predicted physicochemical and pharmacokinetic parameters calculated via SwissADME. (**C**) Bioavailability radar charts where pink areas denote optimal property ranges. Statistical significance (* *p* < 0.05, ** *p* < 0.01) is indicated relative to the control group.

To identify bioactive constituents, seven isolated compounds were evaluated via DPC proliferation assays (Figure 6A) and SwissADME analysis (Figure 6B). Compounds **3** and **6** exhibited the most pronounced bioactivity, increasing DPC proliferation to 155% and 150% of control, respectively, at 40–80 μM (Figure 6A). While compound **1** showed dose-dependent increases and others (**2**, **5**, **7**) showed moderate effects, compound **4** was marginal. SwissADME profiling confirmed that all compounds followed Lipinski’s Rule of Five, suggesting high oral bioavailability (Figure 6B). However, the Veber filter flagged compound **7** as a non-compliant candidate, and the Brenk alert identified compound **6** as potentially unstable or toxic with four structural alerts (Figure 6B). In contrast, atractylenolides **1**–**5** showed moderate solubility, and most atractylenolides fell within the optimal physicochemical space on bioavailability radar charts (Figure 6C). Based on their superior bioactivity, structural stability, and favorable drug-likeness, atractylenolide I (**1**) and III (**3**) were strategically selected for further evaluation (Figure 6C).

### 2.7. Atractylenolides Promote DPC Migration and the Expression of Hair Growth-Associated Factors

**Figure 7:**
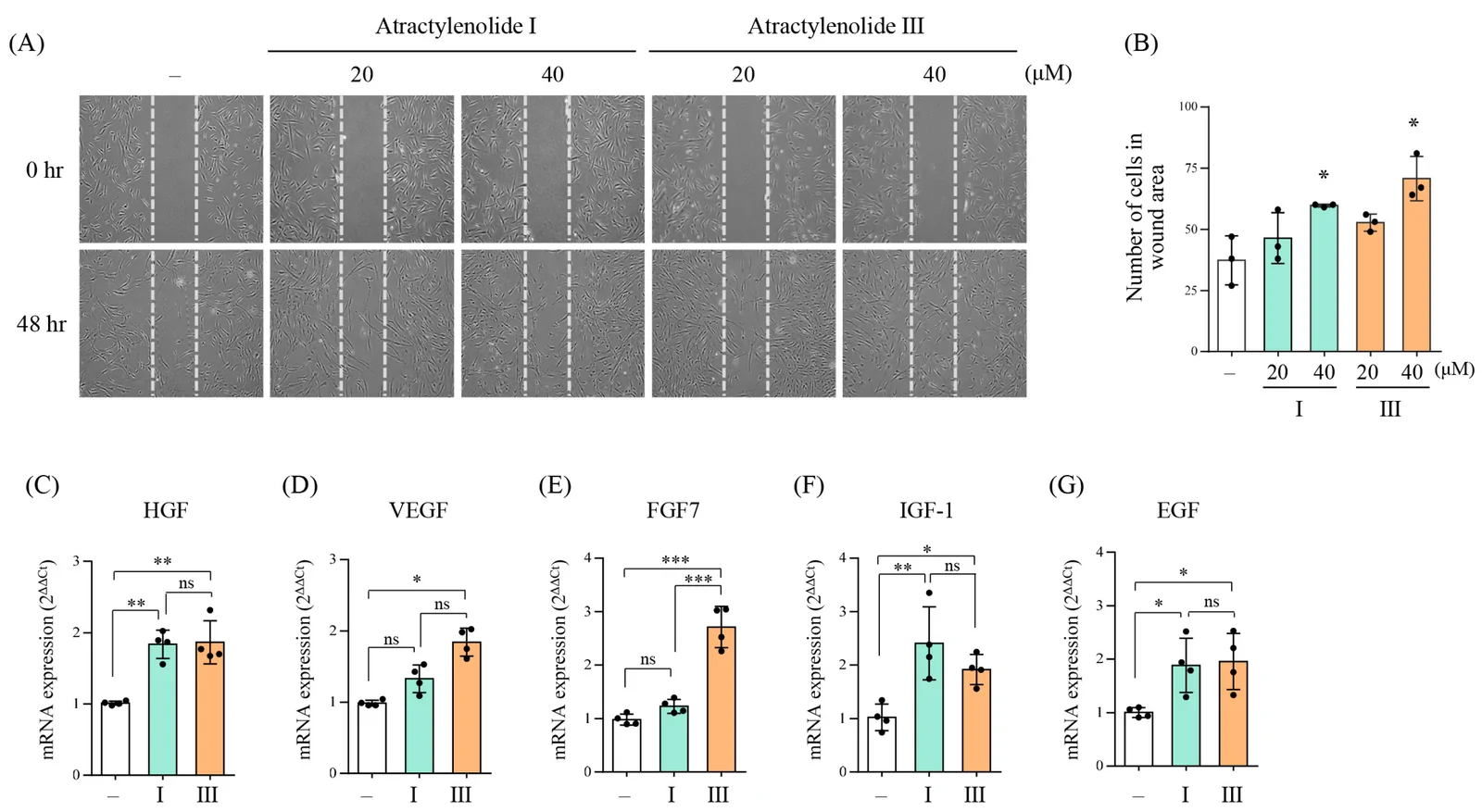
Effects of atractylenolide I and III on DPC migration and hair growth factor expression. (**A**,**B**) Representative images and quantitative analysis of DPC migration after treatment with atractylenolides I and III (20–40 μM) for 48 h under 1% growth factor conditions. (**C**–**G**) RT-PCR analysis of growth-promoting mRNA levels (HGF, VEGF, FGF7, IGF-1, EGF) calculated via the 2^−ΔΔCt^ method. Data represent mean ± S.D (*n* = 3). Statistical significance (* *p* < 0.05, ** *p* < 0.01, *** *p* < 0.001; ns, not significant) is indicated relative to control.

To further elucidate DPC activation by AM-derived constituents, we focused on atractylenolide I and III for functional evaluation. In wound-healing assays, both compounds (20–40 μM) significantly promoted DPC motility compared to controls (Figure 7A,B). Quantitative RT-PCR demonstrated that atractylenolide I and III upregulated distinct subsets of hair growth-promoting genes (Figure 7C–G). Specifically, atractylenolide I increased mRNA levels of HGF, IGF-1, and EGF, whereas atractylenolide III induced robust FGF7 expression along with HGF, VEGF, and IGF-1 (Figure 7C–G). Collectively, these results indicate that atractylenolides potentiate DPC activation by enhancing cellular motility and stimulating transcriptional programs linked to anagen initiation and anagen progression.

## 3. Discussion

In the present study, we systematically demonstrated that AM, a safe food-grade material, accelerates anagen initiation and hair regrowth using DPCs and a mouse model. Through comprehensive analyses, we identified that AM promotes hair follicle cycling associated with GSK3β phosphorylation and β-catenin stabilization.

DPCs are central regulators of follicular morphogenesis and cycling [22]. AM significantly enhanced DPC proliferation and migration under growth factor-limited conditions, suggesting its ability to initiate regenerative signals even in suboptimal microenvironments. Moreover, AM robustly increased the transcription of HGF, VEGF, and FGF7—essential paracrine mediators for angiogenesis and keratinocyte proliferation [23]—thereby reinforcing a growth-promoting niche for anagen transition. Furthermore, our results show that among the two major compounds, atractylenolide III exhibits the growth factor gene expression profile—particularly the robust upregulation of FGF7—most similar to the crude AM extract. The remaining differences in expression patterns strongly suggest that the extract’s overall in vitro efficacy in DPCs is not solely due to atractylenolides I and III, but rather a synergistic effect involving other active constituents, such as polyacetylenes, which collaboratively drive the paracrine signaling associated with β-catenin stabilization in DPCs.

Among seven isolated compounds, atractylenolide I and III were selected for in-depth evaluation based on their superior bioactivity and full compliance with Lipinski’s rule in SwissADME analysis (Figure 6 and Figure 7). These sesquiterpenoids displayed distinct yet overlapping patterns of gene induction: atractylenolide I increased HGF, IGF-1, and EGF, whereas atractylenolide III—closely mirroring the whole AM extract—preferentially enhanced FGF7, HGF, and VEGF (Figure 7).

This comparison suggests that each constituent contributes discrete regulatory roles, while their combined presence in the total extract produces a more integrated transcriptional response. Furthermore, it strongly suggests that the extract’s robust overall efficacy is not solely due to atractylenolides I and III, but rather a synergistic effect involving other active constituents, such as polyacetylenes. Such multi-component interactions, characteristic of botanical extracts, likely explain the potent and coherent biological effects observed with AM in vivo.

Mechanistically, AM modulates the GSK3β/β-catenin signaling axis, a canonical pathway governing anagen activation. AM increased inhibitory phosphorylation of GSK3β (Ser9), stabilizing β-catenin in DPCs and hair follicles (Figure 2 and Figure 5). This process was correlated with the activation of Akt, ERK, and p38, which converge via direct and indirect phosphorylation to reduce GSK3β activity [24]. Stabilized β-catenin subsequently acts as a critical transcriptional driver of anagen entry [25]. Given that atractylenolides influence PI3K/Akt and MAPK pathways [26], AM likely coordinates these signaling cascades—potentially through growth factor-driven mechanisms—to promote anagen entry.

In vivo, oral AM promoted active anagen progression by increasing dermal thickness, hair shaft diameter, and follicular growth/maturation (Figure 3 and Figure 4). Notably, the absence of significant changes in body weight or liver mass highlights its favorable safety profile (Figure 3B,C). Given its status as an officially recognized edible material, these findings support further investigation of AM as a candidate for modulating physiological hair-cycle progression.

Importantly, our in vivo model reflects physiological hair cycle progression rather than the complex etiology of clinical alopecia. Therefore, the therapeutic efficacy of AM for specific human conditions, such as androgenetic alopecia, and the in vivo functional efficacy of pure atractylenolide single compounds remain to be rigorously verified using appropriate disease-specific pathological models in the future.

A few considerations remain. First, the synchronized depilation model differs from asynchronous human hair cycling, potentially affecting temporal responsiveness in humans. Second, although AM activates Akt/ERK/p38-mediated GSK3β signaling via Ser9 phosphorylation, the evaluation of active Tyr216 phosphorylation was not performed, leaving the comprehensive regulatory mechanism of GSK3β partially explored. Third, while we observed increased growth factor mRNA expression in DPCs, this does not demonstrate actual protein secretion or functional paracrine activity on surrounding epithelial follicular cells. Fourth, our in vitro evaluations are restricted to a DPC monoculture system, which cannot reproduce the reciprocal epithelial–mesenchymal signaling occurring within the intact follicle; thus, future studies using 3D co-culture systems or intact human hair follicle organ cultures are required to validate these downstream follicular responses. Fifth, although AM stimulates the Akt, ERK, p38, and β-catenin pathways, the definitive causal requirement of each kinase and signaling node remains to be formally established using appropriately controlled pharmacological or genetic approaches. Finally, while Ki-67 and β-catenin accumulation was observed in the hair matrix compartment, the direct activation, expansion, or differentiation of bulge stem cells was not directly established using compartment-specific stem cell markers (e.g., CD34, LGR5). These boundaries do not invalidate our current findings; rather, they are essential for accurately defining the scope of our study. Furthermore, comprehensive long-term systemic safety studies remain to be conducted in the future.

In summary, our findings demonstrate that AM enhances DPC-associated pro-growth signaling and significantly accelerates anagen entry following depilation by stabilizing β-catenin via mechanisms consistent with the involvement of the Akt/ERK/p38 pathways. While validation in pathological models and human ex vivo systems is strictly required before considering clinical relevance for specific alopecia disorders, the current findings support further investigation of AM as a candidate for modulating physiological hair-cycle progression.

## 4. Materials and Methods

### 4.1. Cells, Reagents and Antibodies

Primary human follicle dermal papilla cells (DPCs), CEFOgro-HDP Basal Medium, growth factor cocktails, and penicillin-streptomycin mixture were purchased from CEFObio (Seoul, Republic of Korea). Cell Counting Kit-8 (CCK-8) reagent was purchased from Dojindo (Kamimashiki, Kumamoto, Japan). Dimethyl Sulfoxide (DMSO), Accutase solution, iso-propanol, chloroform were purchased from Sigma-Aldrich Co. (St. Louis, MO, USA). Fetal bovine serum (FBS) was purchased from Gibco (Grand Island, NY, USA). BCA protein assay kit, DEPC-water, Alexa fluor 488 (A-11001) and Hoechst (33342) were purchased from Thermo Fisher Scientific Inc (Waltham, MA, USA). Polyvinylidene difluoride (PVDF) membrane was purchased from Merck Millipore (Billerica, MA, USA). β-catenin (#8480), p-Akt (#9270), Akt (#9272), p-GSK3β (#9232), GSK3β (#5676), p-ERK (#4370), and ERK (#4695) and Ki-67 (#9129) antibodies were purchased from Cell Signaling Technology, Inc. (Danvers, MA, USA). p-p38 (sc-166182), p38 (sc-7149) and β-actin (sc-47778) antibodies were obtained from Santa Cruz Biotechnology (CA, USA). HRP-conjugated goat anti-rabbit IgG and HRP-conjugated goat anti-mouse IgG were purchased from Enzo (Farmingdale, NY, USA). Reagents for cDNA synthesis were purchased from QIAGEN (Hilden, North Rhine-Westphalia, Germany).

### 4.2. Extract Preparation, and Constituent Compounds

The rhizomes of *A. macrocephala* used in this study were obtained from the National Institute of Forest Science (Yeongju, Republic of Korea) and authenticated by the Herbarium of the College of Pharmacy at Chungbuk National University, where a voucher specimen (CBNU2023-AM) has been deposited. Preparation of the *A. macrocephala* extract (AM) followed the previously described method [16], yielding a crude methanolic extract used in this study. In addition, our prior phytochemical investigation of AM isolated seven major constituents, atractylenolide I (**1**), atractylenolide II (**2**), atractylenolide III (**3**), atractylenolide IV (**4**), atractylenolide VII (**5**), 14-acetoxy-12-senecioyloxytetradeca-2*E*,8*E*,10*E*-trien-4,6-diyn-1-ol (**6**), and 14-acetoxy-12-methylbutyl-2*E*,8*E*,10*E*-trien-4,6-diyn-1-ol (**7**).

### 4.3. Cell Culture

DPCs were maintained in CEFOgro-HDP Basal Medium supplemented with 4% (*v*/*v*) growth factor cocktail and 0.5% penicillin-streptomycin mixture in a humidified atmosphere at 37 °C with 5% CO_2_. Cells were seeded at a density of 5 × 10^5^ cells/mL onto 100 mm culture dishes and subcultured every 2–3 days. To ensure the retention of hair-inducing capacity and phenotypic characteristics, only early-passage cells (passages 4 to 10) were used for all experiments. For experiments under growth factor–deficient conditions, the cocktail concentration was reduced to 1%.

### 4.4. Cell Proliferation Assay

DPCs were seeded in 96-well plates at 1 × 10^3^ cells/well and incubated overnight in 1% growth factor medium. Cells were then treated with AM (15, 30, 60, 120 μg/mL) or isolated compounds (40 and 80 μM) for 48 h. The positive control consisted of 4% growth factor medium. Cell proliferation was assessed using the CCK-8 assay according to the manufacturer’s instructions. Absorbance at 450 nm was measured using a microplate reader (Epoch, Biotek, Winooski, VT, USA).

### 4.5. Wound Healing (Migration) Assay

DPCs were seeded in 6-well plates at 1 × 10^5^ cells/well and incubated overnight in 1% growth factor medium. A scratch was created in the center of each well using a sterile cell scratcher (SPL, Pocheon, Republic of Korea), and detached cells were removed by washing with PBS. Cells were then treated with AM or compounds in 1% growth factor medium for 48 h. Images were captured under a phase-contrast microscope (ECLIPSE TS2, Nikon, Tokyo, Japan). Migrated cells were quantified using ImageJ software (version 1.54, NIH, Bethesda, MD, USA).

### 4.6. RNA Extraction and Quantitative RT-PCR

Total RNA was extracted using NucleoZOL (BMS, Seoul, Republic of Korea) and precipitated with isopropanol. RNA quantity and purity were measured using a NanoDrop spectrophotometer (Thermo Fisher Scientific, Waltham, MA, USA). cDNA was synthesized from 2 ng RNA using the Omniscript RT kit (QIAGEN, Hilden, Germany). Quantitative PCR was performed with 2 μL cDNA using SYBR Green Master Mix (Bio-Rad, Hercules, CA, USA) on a QuantStudio 1 Real-Time PCR system (Applied Biosystems, Waltham, MA, USA). Cycling conditions were: 95 °C for 15 s, 60 °C for 60 s, and 95 °C for 15 s for 40 cycles. Relative mRNA levels were calculated using the 2^−^^ΔΔCT^ method with β-actin as the internal control. Primer sequences are listed in Table 1.

### 4.7. Protein Extraction and Western Blot Analysis

Cells were lysed in CETI buffer (Translab, Daejeon, Republic of Korea) on ice for 15 min, followed by centrifugation at 13,000 rpm for 15 min at 4 °C. Protein concentrations were determined using BCA assay. Equal amounts of protein were separated by 10% SDS-PAGE and transferred to PVDF membranes. Membranes were blocked with 5% skim milk in TBS-T for 1 h at room temperature and incubated overnight at 4 °C with primary antibodies. After washing, membranes were incubated with HRP-conjugated secondary antibodies for 1 h at room temperature. Bands were visualized using ECL (Thermo Fisher Scientific) and imaged with an Amersham Imager 680 (Cytiva, Marlborough, MA, USA). Densitometry was performed using Multi Gauge Version 3.0 (Fujifilm, Tokyo, Japan).

### 4.8. Time-Synchronized Depilation Mouse Model and AM Administration

Animal experiments were approved by the Institutional Animal Care and Use Committee of the Korea Research Institute of Bioscience and Biotechnology (KRIBB-AEC-25099) and conducted in strict accordance with the relevant guidelines and regulations. Six-week-old male C57BL/6N mice were obtained from Koatech (Pyeongtaek, Republic of Korea) and acclimated for 1 week. Mice were maintained under standard conditions (22 ± 3 °C, 50 ± 20% humidity, 12 h light/dark cycle) with ad libitum access to food and water. Mice were anesthetized by intraperitoneal injection of avertin (2,2,2-tribromoethanol; Sigma-Aldrich, MO, USA), and the dorsal skin was shaved with a clipper and subsequently treated with a depilatory agent (Veet; Reckitt Benckiser, Slough, UK) to completely remove remaining hair. After 24 h of skin stabilization, mice in telogen phase were randomly assigned to vehicle (saline, 200 μL), AM low dose (30 mg/kg), AM high dose (60 mg/kg), minoxidil (30 mg/kg) groups (*n* = 6 per group). Treatments were administered orally daily for 3 weeks.

### 4.9. Hair Growth Assessment and Hair Shaft Analysis

Hair regrowth on the dorsal skin was visually monitored every 2–3 days using an iPhone 16 Pro smartphone camera (Apple, Cupertino, CA, USA). For hair shaft analysis, newly grown hairs were collected from randomly selected mice in each group at the end of the experiment. Hair length was measured using a digital vernier caliper. Hair diameter and cuticle morphology were examined using scanning electron microscopy (SEM; HITACHI REGULUS 8100, Hitachi, Tokyo, Japan). Briefly, hairs were mounted on SEM stubs with conductive adhesive, sputter-coated with gold, and imaged at multiple magnifications. Hair diameter was measured using the built-in measurement software (version 3.8, Hitachi High-Tech Corp.) of the SEM system.

### 4.10. Histological and Immunofluorescence Analysis

Dorsal skin samples were fixed in 10% neutral buffered formalin, embedded in paraffin, and sectioned at 5 μm. Sections were stained with hematoxylin and eosin (H&E) for morphological evaluation. Dermal thickness and follicle counts were quantified using ImageJ. For immunofluorescence, deparaffinized sections were subjected to antigen retrieval and incubated with primary antibodies against β-catenin and Ki-67 overnight at 4 °C, followed by Alexa Fluor-conjugated secondary antibodies. Nuclei were counterstained with DAPI. Fluorescent images were captured using a confocal microscope (3DHISTECH, Pannoramic Scan II Digital Scanner, Budapest, Hungary).

### 4.11. In Silico Prediction of Physicochemical and Pharmacokinetic Properties

The physicochemical properties and pharmacokinetic potential of the seven isolated compounds (**1**–**7**) were predicted using the SwissADME web tool (http://www.swissadme.ch; (accessed on 23 December 2025)) by inputting the Simplified Molecular Input Line Entry System (SMILES) strings of each chemical structure.

### 4.12. Statistical Analysis

All data are presented as the mean ± standard deviation (SD) from at least three independent experiments (*n* = 3). Statistical significance was determined using one-way analysis of variance (ANOVA), followed by Dunnett’s post-hoc test for multiple comparisons between the control and treated groups. For quantitative real-time PCR, relative mRNA expression levels were calculated using the 2^−ΔΔCt^ method. Differences were considered statistically significant at *p*-values of less than 0.05 (* *p* < 0.05), 0.01 (** *p* < 0.01), or 0.001 (*** *p* < 0.001). Values with *p* > 0.05 were considered not significant (ns).

## Figures and Tables

**Table 1:** Information of primers used in this study.

Gene	Sequences (Human)	Product Size (bp)	GeneBank Accession No.
*VEGF*	5′-TTGCCTTGCTGCTCTACCTCCA-3′	126	NM_001171623.2
	5′-GATGGCAGTAGCTGCGCTGATA-3′		
*HGF*	5′-GAGAGTTGGGTTCTTACTGCACG-3′	102	NM_000601.6
	5′-CTCATCTCCTCTTCCGTGGACA-3′		
*EGF*	5′-TGTAGCGGTTGTTCCTCACC-3′	295	NM_001963.6
	5′-TCAGGGCTGTATGGGCAAAG-3′		
*IGF-1*	5′-CTCTTCAGTTCGTGTGTGGAGAC-3′	134	NM_001414007.1
	5′-CAGCCTCCTTAGATCACAGCTC-3′		
*FGF7*	5′-GGGACCCAAGAGATGAAGAA-3′	73	NM_002009.4
	5′-TTCACTTTCCACCCCTTTGA-3′		
*β-actin*	5′-CACCATTGGCAATGAGCGGTTC-3′	140	NM_001101.1
	5′- AGGTCTTTGCGGATGTCCACGT -3′		

## Data Availability

The data presented in this study are available on request from the corresponding authors.

## Abbreviations

The following abbreviations are used in this manuscript:

AM	*Atractylodes macrocephala* rhizoma extract
Akt	Protein kinase B
CCK-8	Cell Counting Kit-8
DAPI	4′,6-diamidino-2-phenylindole
DHT	dihydrotestosterone
DMSO	Dimethyl Sulfoxide
DPCs	dermal papilla cells
EGF	epidermal growth factor
ERK	extracellular signal-regulated kinase
FBS	fetal bovine serum
FDA	Food and Drug Administration
FGF7	fibroblast growth factor 7
GSK3β	glycogen synthase kinase 3 beta
H&E	hematoxylin and eosin
HGF	hepatocyte growth factor
IGF-1	insulin-like growth factor 1
MFDS	Ministry of Food and Drug Safety
PVDF	polyvinylidene difluoride
RT-PCR	reverse transcription polymerase chain reaction
SDS-PAGE	sodium dodecyl sulfate-polyacrylamide gel electrophoresis
SEM	scanning electron microscopy
VEGF	vascular endothelial growth factor
